# Autoimmune Thrombocytopenia in Pregnancy: Insights from an Uncommon Case Presentation and Mini-Review

**DOI:** 10.3390/jcm14030872

**Published:** 2025-01-28

**Authors:** Andrei Mihai Malutan, Oana Teodora Pascu, Doru Diculescu, Razvan Ciortea, Ligia Blaga, Renata Nicula, Carmen Bucuri, Maria Roman, Ionel Nati, Cristina Mihaela Ormindean, Viorela Suciu, Dan Mihu

**Affiliations:** 1 2nd Department of Obstetrics and Gynecology, ‘Iuliu Hatieganu’ University of Medicine and Pharmacy, 400006 Cluj-Napoca, Romania; malutan.andrei@gmail.com (A.M.M.); diculescu@yahoo.com (D.D.); r_ciortea@ymail.com (R.C.); rentanicula@yahoo.com (R.N.); cbucuri@yahoo.com (C.B.); mpr1387@gmail.com (M.R.); nati.ionel@yahoo.com (I.N.); cristina.mihaela.podan@gmail.com (C.M.O.); suciuviorela@yahoo.com (V.S.); dan.mihu@yahoo.com (D.M.); 2 Department of Neonatology, ‘Iuliu Hatieganu’ University of Medicine and Pharmacy, 400006 Cluj-Napoca, Romania; blaga.ligia@yahoo.com; 3 Clinical Department of Surgery, “Constantin Papilian” Emergency Clinical Military Hospital, 22 G-ral Traian Mosoiu, 400132 Cluj-Napoca, Romania

**Keywords:** thrombocytopenia, autoimmune, pregnancy, diagnostic

## Abstract

Thrombocytopenia, defined as a platelet count below 150 × 10^9^/L, is the second most common hematological abnormality after anemia found among European women in the third trimester of pregnancy. Most of the cases are mild, asymptomatic, and diagnosed accidentally. The primary causes of thrombocytopenia are linked to the pregnancy itself and include gestational thrombocytopenia (GT), autoimmune thrombocytopenia (ITP), and pre-eclampsia or HELLP syndrome-associated thrombocytopenia. First-line therapies for ITP include corticosteroids and intravenous immunoglobulin (IVIG). We came across a case of severe thrombocytopenia (platelet count of 9 × 10^9^/L) associated with severe anemia (Hb 5.9 g/dL) at 30 weeks of gestation, with no personal or family history of bleeding disorders. A comprehensive hematologic, infectious, and rheumatological workup was performed to narrow the diagnosis. Despite aggressive corticosteroid therapy and immunoglobulin treatment, the patient’s thrombocytopenia persisted, imposing delivery at 34 weeks. This article highlights the complex presentation and management of severe thrombocytopenia and anemia during pregnancy.

## 1. Introduction

Thrombocytopenia, defined as a platelet count below 150 × 10^9^/L, can be divided into mild, 100–150 × 10^9^/ L; moderate, 50–100 × 10^9^/L; and severe, less than 50 × 10^9^/L. The International Working Group established a platelet threshold of less than 100 × 10^9^/L as clinically relevant thrombocytopenia [1,2,3].

The etiology of thrombocytopenia in pregnant women can be categorized into two groups: pregnancy-associated causes and causes independent of pregnancy. Pregnancy-related conditions account for over 90% of thrombocytopenia cases, including GT (70–80%), pre-eclampsia (15–20%), HELLP syndrome (hemolysis, elevated liver enzymes, low platelets) (<1%), and acute fatty liver of pregnancy (<1%). Independent causes are varied and can be either congenital, such as von Willebrand syndrome and hereditary thrombocytopenia, or acquired. Acquired causes include immune-mediated conditions like ITP, systemic lupus erythematosus, antiphospholipid syndrome, drug-induced thrombocytopenia, thrombotic thrombocytopenic purpura (TTP), and hemolytic–uremic syndrome (HUS). Non-immune-mediated causes include secondary thrombocytopenia related to infectious diseases, e.g., human immunodeficiency virus (HIV), hepatitis C virus (HCV), Epstein–Barr virus (EBV), bone marrow disorders (acute leukemia), poor nutrition, folate or vitamin B12 deficiency, and hypersplenism. Even if the etiology is complex, 90% of cases are due to pregnancy-related conditions, of which GT and ITP are the most common. It is important to emphasize that there are no characteristic signs or symptoms and both are exclusion diagnoses [4,5,6,7,8].

ITP affects roughly 1 in every 1000 to 10,000 pregnancies. It can be categorized as either primary or secondary, depending on other underlying health conditions at the time of diagnosis, such as HCV, HIV, or lymphoproliferative disorders. Additionally, ITP can be further classified according to the timing of onset: newly diagnosed (within the first 3 months after diagnosis), persistent (between 3 and 12 months), or chronic (lasting 12 months or more). It is the most common cause of a platelet count below 50 × 10^9^/L in the first and second trimesters. Platelet counts may decrease during gestation, and 15% to 35% of mothers require treatment before labor and delivery. Maternal and neonatal outcomes are generally favorable, so ITP is not typically considered a contraindication to pregnancy [9,10,11].

The diagnosis of ITP is predominantly established by excluding other potential causes of thrombocytopenia. This process involves a comprehensive assessment of the patient’s medical history, a detailed physical examination, a complete blood count (CBC) analysis, and a peripheral blood film to rule out other hematological disorders, such as hereditary thrombocytopenia and pseudo thrombocytopenia. Sometimes, the diagnosis may only be confirmed retrospectively based on the response to ITP-specific treatments [3,4,12,13,14]

Typically, patients with mild or moderate thrombocytopenia remain asymptomatic, whereas those with severe thrombocytopenia, particularly with platelet counts between 30 and 50 × 10^9^/L, may experience excessive bleeding from trauma, or, in rare cases, present with purpura. When platelet counts are between 10 and 30 × 10^9^/L, bleeding can occur even with minimal trauma, and counts of less than 10 × 10^9^/L are associated with an increased risk of spontaneous bleeding and petechiae [4,15].

The pathological mechanism of ITP involves an accelerated destruction of platelets opsonized by anti-platelet autoantibodies primarily targeting platelet membrane glycoproteins. This process occurs through the reticuloendothelial systems in the spleen and other organs. Additionally, these autoantibodies cause the failure of megakaryocyte maturation and trigger apoptosis, leading to a reduction in platelet production. Besides anti-platelet autoantibodies, immune complexes, complement, and cytotoxic T cells may also contribute to the development of thrombocytopenia [15,16,17].

The primary treatments for maternal ITP are corticosteroids and intravenous immunoglobulin (IVIG). The choice of therapy should be tailored to the individual patient, considering the urgency and duration of the needed platelet increase and the potential side effects [3,4,5,18,19,20]. Corticosteroids are the standard initial treatment, typically administered for up to 21 days. However, there is no established evidence to guide the sequencing of therapies for patients with recurrent or persistent thrombocytopenia following an initial course of treatment. Response to corticosteroid therapy usually occurs within 4–14 days, with maximum effects seen within 1–4 weeks. The maternal risks of corticosteroid use include exacerbating gestational diabetes, hypertension, osteoporosis, weight gain, and psychosis. Relevant contraindications to corticosteroid therapy include insulin-dependent or uncontrolled diabetes, psychiatric disorders, and active infection. High doses may pose risks to the fetus, including the premature rupture of membranes, adrenal suppression, and a slight increase in the incidence of cleft lip if used in the first trimester [1,3]. In cases refractory to corticosteroids, IVIG is initiated, particularly when significant adverse effects arise from corticosteroids or when a more rapid increase in platelet count is needed. IVIG typically produces an initial response within 1–3 days, with maximum effects achieved within 2–7 days. Splenectomy is generally avoided during pregnancy due to fetal risks and technical challenges, especially in the later stages of gestation [1,2,3]. Platelet transfusions are reserved for urgent situations, such as preparation for emergency surgery or the management of life-threatening hemorrhage. Other therapeutic options, including cytotoxic agents (e.g., cyclophosphamide or vinca alkaloids), Rh D immunoglobulin, or immunosuppressive agents (e.g., azathioprine or rituximab), have not been sufficiently studied during pregnancy and may pose potential risks to the fetus [1,2,3].

Thrombocytopenia in infants born to mothers with ITP is generally mild to moderate and occurs in up to 20% of cases [14]. However, affected infants require close monitoring and, in some cases, therapeutic intervention. Although rare, intracranial hemorrhage occurs in 1% to 3% of cases and represents a serious complication. Postnatal management frequently involves the use of IVIG and low-dose steroid therapy with a close follow-up after discharge from the hospital. Platelet transfusion is recommended for a platelet count below 100 × 10^9^/L if there is active bleeding, for a platelet count between 30 and 49 × 10^9^/L if the patient is unstable, for infants with a birth weight under 1500 g and ≤7 days old, before surgery, and in all cases with a platelet count below 30 × 10^9^/L [21,22,23].

## 2. Case Report

A 27-year-old primigravida presented at 30 weeks of gestation with severe anemia (Hb 5.9 g/dL) and severe thrombocytopenia (platelet count 9 × 10^9^/L). Her prenatal history was notable for a platelet count of 13 × 10^9^/L at 10 weeks of gestation, along with mild anemia (Hb 10.6 g/dL). All the investigations, abnormal results, and treatments are summarized in Table 1. During the pregnancy, the patient reported recurrent episodes of epistaxis and a single episode of purpura on her lower limbs. She had no personal or family history of bleeding disorders. On physical examination, her blood pressure was normal, and there were no signs of bruising, petechiae, or other bleeding manifestations. Initial laboratory tests revealed severe anemia (Hb 4.9 g/dL) and thrombocytopenia (platelet count <10 × 10^9^/L). The patient was transfused with 2 units of red blood cells (RBc), and dexamethasone therapy was initiated to accelerate fetal lung maturation.

### 2.1. Hematologic Evaluation

The following day, a hematology consultation confirmed severe anemia (Hb 7.5 g/dL) and thrombocytopenia (platelet count < 9 × 10^9^/L). A peripheral blood smear showed anisocytosis, macrocytes, rare microcytes, ovalocytes, rare dacrocytes, and some polychromatophilic macrocytes. The antiglobulin test (Direct Coombs Test) was negative and LDH was within normal limits. TTP, HUS, and HELLP syndrome were excluded based on clinical and laboratory findings. The working diagnosis was ITP associated with macrocytic anemia. The hematologist recommended continuing treatment with 12 mg/day of dexamethasone, along with further transfusions as needed when Hb fell below 7 g/dL.

Despite severe thrombocytopenia throughout her six-week hospitalization, the patient displayed no hemorrhagic manifestations, an atypical finding that complicates the usual clinical presentation of ITP. After excluding life-threatening conditions and given that the patient had experienced severe thrombocytopenia since the first trimester, a provisional diagnosis of ITP was established.

### 2.2. Further Investigations

Additional tests were performed to investigate the etiology of thrombocytopenia. Hematological and autoimmune workups, including testing for anticardiolipin antibodies, antinuclear antibodies (ANA), anti-RNP, anti-Ro, anti-dsDNA, anti-LKM, anti-MPO (p-ANCA), anti-PR3 (c-ANCA), anti-smooth muscle antibodies, and complement components C3 and C4, were all within normal limits. Immunoglobulin levels (IgA, IgG, and IgM) were also normal. Further blood tests were conducted to investigate the anemia, which excluded hemolysis and showed normal folate levels, slightly low vitamin B12, and normal ferritin levels. Vitamin B12 and folic acid supplementation were initiated.

Due to the presence of thrombocytopenia associated with megaloblastic anemia, normal levels of folate, and just slightly low levels of vitamin B12, other causes of megaloblastic anemia, like bone marrow disorders, hypothyroidism, alcoholism, medication, and liver disease needed to be ruled out.

### 2.3. Bone Marrow Biopsy and Infectious Workup

Due to the persistent thrombocytopenia (platelet count < 15 × 10^9^/L) and an unclear anemia etiology, a bone marrow biopsy was performed. The biopsy revealed the anemia was due to a deficiency in maturation factors, likely related to iron, and the thrombocytopenia was suggestive of a peripheral mechanism (megakaryocytic series were present but quantitatively reduced, possibly due to technical reasons).

An infectious disease consultation excluded clinical signs of infection. Screening for HIV, HBV, cytomegalovirus (CMV), and parvovirus returned negative results.

### 2.4. Ultrasound Findings

Multiple ultrasound examinations during hospitalization demonstrated normal fetal development. We also assessed the peak systolic velocity (PSV) in the middle cerebral artery to estimate fetal hemoglobin and identified levels ranging from 10.6 g/dL to 16.1 g/dL.

### 2.5. Delivery, Newborn, and Postpartum

It is important to note that there is no national guideline for the management of autoimmune thrombocytopenia in pregnancy. The approach to this complex case was guided by international recommendations and the expertise of a multidisciplinary team, which included specialists in obstetrics, hematology, neonatology, anesthesia, infectious diseases, and rheumatology. The primary objective was to develop an intervention plan that prioritized the safety of both the mother and the fetus. To achieve this, rigorous monitoring of hematological parameters was conducted, and the therapeutic plan was continuously adjusted to optimize the timing of delivery.

At 34 weeks of gestation, following a unanimous decision with the neonatology department, an elective cesarean section was scheduled due to prolonged corticosteroid therapy and the increasing risks associated with continuing the pregnancy. Dexamethasone was gradually tapered, and IVIG (0.4 g/kg/day) was administered two days before the cesarean section and on the day of the procedure. Despite these interventions, the patient’s platelet counts remained below 15 × 10^9^/L.

The cesarean section, performed under general anesthesia, was preceded by the transfusion of one unit of leuko-reduced RBc and one unit of cryoprecipitate. The surgery proceeded without complications, and no bleeding incidents occurred.

The newborn was a female, weighing 2800 g, with an Apgar score of 8 at birth and 9 at 5 min post-delivery. Paraclinical examinations showed mild anemia (Hb 14.3 g/dL) and moderate thrombocytopenia (platelet count 76 × 10^9^/L) on the first day of life. IVIG (0.5 g/kg/day) was administered on the first and second days of life, with platelet counts normalizing by the third day. No hemorrhagic complications occurred. The mild anemia persisted without progression during the hospitalization.

Post-delivery, the patient’s platelet counts initially increased to 28 × 10^9^/L but gradually decreased to 19 × 10^9^/L before discharge.

### 2.6. Postpartum Follow-Up

One month postpartum, the patient’s platelet count had increased to 38 × 10^9^/L, with an Hb of 8.3 g/dL and a leukocyte count of 3.87 × 10^9^/L. At three months postpartum, laboratory tests revealed pancytopenia, with an Hb of 10.8 g/dL, platelet count of 20 × 10^9^/L, and leukocyte count of 3.32 × 10^9^/L, accompanied by neutropenia. No data were available regarding the laboratory results of the newborn after discharge or those of the mother beyond three months post-discharge.

## 3. Discussion

We present a case of a pregnant patient associating with severe thrombocytopenia, without any significant clinical manifestations such as bleeding or purpura, adding increased complexity to the typical presentation of severe thrombocytopenia during pregnancy. Several studies have similarly documented cases where patients with severe thrombocytopenia did not exhibit clinical bleeding manifestations, aligning with our findings [4,15].

While ITP is typically characterized by isolated thrombocytopenia, it can occasionally be associated with other hematological abnormalities, such as bone marrow dysfunction or vitamin B12 and folate deficiencies. A notable aspect of this case was the coexistence of thrombocytopenia with macrocytic anemia, for which the patient required multiple erythrocyte transfusions, without bone marrow dysfunction or vitamin B12 or folate deficiencies [15,16,17,18].

Guidelines for the investigation and management of autoimmune thrombocytopenia in pregnancy are outlined in the American Society of Hematology (ASH) clinical practice guidelines published in 2011 and the American College of Obstetricians and Gynecologists (ACOG) guidelines from 2019. However, the 2019 ASH guidelines on ITP do not address pregnancy-specific considerations. The Belgian guidelines for the diagnosis and management of primary ITP, published in 2021, categorize diagnostic investigations into three tiers: “Basic evaluation”, “Tests of potential utility”, and “Tests of unproven utility”. This classification aligns with the framework presented in the “International Consensus Report on the Investigation and Management of Primary Immune Thrombocytopenia” by Provan et al. Similarly, consensus guidelines for the management of adult ITP in Australia and New Zealand recommend a comparable range of diagnostic investigations [1,3,24,25].

In both our case and the relevant literature, the diagnosis of ITP was established after excluding other potential causes of isolated thrombocytopenia. This is consistent with the standard diagnostic approach, which includes evaluating the patient’s medical history, physical examination, and CBC, followed by the exclusion of other hematologic conditions through peripheral blood smears. Just as the investigations recommend further autoimmune and hematological workups, our case involved testing for a wide range of autoimmune markers, including ANA, ACA, and complement levels, all of which returned normal results [1,7,26,27].

The comprehensive range of investigations undertaken adhered to the recommendations of national and international associations, encompassing tests categorized as “Basic evaluation”, “Tests of potential utility”, and “Tests of unproven utility”. This thorough diagnostic approach ensured a definitive diagnosis.

Corticosteroids were initiated as the first-line therapy, administered following international guideline recommendations, which advocate for their use at the minimum effective dose. Due to a suboptimal response to corticosteroids, treatment was transitioned to IVIG. Despite aggressive treatment with corticosteroids and IVIG, the patient’s thrombocytopenia persisted. To achieve a platelet count sufficient for safe cesarean delivery, a platelet transfusion was required. In cases of severe thrombocytopenia, as seen in our patient, administering a platelet transfusion before surgery is a common practice to reduce the risk of intraoperative bleeding. Research supports the idea that perioperative platelet transfusions effectively elevate platelet counts and mitigate bleeding risks in patients with extremely low platelet levels, often below 10 × 10^9^/L [7,27,28]. The transfusions successfully elevated platelet levels enough to safely proceed with the surgery without bleeding complications, which is consistent with the findings from several studies. This stepwise approach of combining dexamethasone, IVIG, and platelet transfusion follows the standard of care for severe thrombocytopenia in pregnancy and aims to balance efficacy with the safety of both the mother and fetus. Second-line therapies such as thrombopoietin-receptor agonists were not recommended by the hematologist, as their use lacks a national consensus and sufficient safety data in pregnant women. Immunosuppressive agents were also not recommended by the hematologist, reflecting a cautious approach due to potential risks in pregnancy [1,3,7].

The treatment recommendations in the 2019 ACOG guidelines are summarized as follows: first-line therapy consists of corticosteroids, IVIG, or a combination of both. The treatment protocol in this case followed the recommendations outlined in the international guidelines for ITP [1,3,7,10].

Notably, despite the patient’s severe thrombocytopenia and anemia, the cesarean delivery was not associated with any bleeding complications. This is particularly significant given the typically increased bleeding risk in patients with severe thrombocytopenia, especially during major surgical interventions like a cesarean section. The absence of associated hemorrhagic events may point to the effectiveness of perioperative platelet transfusions in maintaining adequate hemostasis during delivery.

Both the literature and our case support the notion that most cases of neonatal thrombocytopenia secondary to maternal ITP are manageable with IVIG and that significant bleeding complications, such as intracranial hemorrhage, are rare. Our case aligns with the general trend in the literature, where thrombocytopenia has been successfully managed with IVIG without the need for additional interventions such as steroid therapy, and no severe complications developed during hospitalization [21,22,23].

Postnatal management involves close monitoring of the patient, regular consultations with the multidisciplinary team, and adjustments to the therapeutic plan based on the patient’s clinical condition and laboratory results. This approach aligns with international recommendations for the management of autoimmune thrombocytopenia. Effective communication and collaboration among specialists are critical to ensuring optimal care [1,2,3].

A rare but significant complication in such cases is the failure of platelet counts to recover after delivery. In our case, the patient’s thrombocytopenia persisted postpartum, and by three months, she had developed pancytopenia, complicating her clinical course. The development of pancytopenia postpartum in ITP patients is unusual and suggests a more complex underlying condition. Further investigation, such as a repeat bone marrow biopsy or additional autoimmune and infectious workups, may be required to rule out other potential causes. Comparatively, classic ITP typically involves a singular thrombocytopenia that resolves or stabilizes after delivery, making the persistence of cytopenia in our patient a significant deviation from the expected disease course [29,30,31,32,33].

This comprehensive, protocol-driven strategy facilitated a favorable outcome for both the mother and the newborn, despite the challenges posed by a severe hematological condition.

The current study has a series of limitations, detailed as follows: (1) the lack of consensus regarding second-line therapy for ITP remains a significant challenge; and (2) the absence of follow-up data concerning the newborn after discharge, as well as the unavailability of further information due to the patient’s decision to discontinue post-discharge monitoring and treatment.

## 4. Conclusions

In conclusion, the management of thrombocytopenia during pregnancy poses significant challenges due to several factors, including the limited time available for diagnosis and treatment, the need to consider the condition of the fetus, and the potential impact of treatment on fetal health. Additionally, maintaining a hemostatic platelet count is crucial, particularly in preparation for invasive procedures. The primary goal in managing thrombocytopenia in pregnancy is not to achieve normal platelet levels but to maintain a platelet count that ensures safety.

In this case, we presented a patient with severe thrombocytopenia, yet without clinical manifestations. The primary focus was to secure a safe platelet count for delivery. Due to the patient’s lack of response to standard treatment, a platelet transfusion was administered to enable a safe cesarean section.

The unique aspects of this case include the absence of hemorrhagic symptoms despite severe thrombocytopenia, the inadequate response to standard treatment, and, most notably, the failure of platelet counts to recover post-delivery.

The transition from isolated thrombocytopenia to pancytopenia is less common, but it highlights the complexity of managing autoimmune conditions that affect multiple blood cell lines. Regular monitoring and adjusting of therapeutic approaches are crucial for managing these cases.

## Figures and Tables

**Table 1:** Investigation results and associated treatments during pregnancy.

Timeline	Investigations	Abnormal Results	Treatment
**ADMISSION** 30 WG	CBC	Severe anemia (Hb 5.9 g/dL); severe thrombocytopenia (PCT 9 × 10^9^/L)	Dexamethasone; RBc
30–33 WG	CBC, peripheral blood smear, vitamin B12, folic acid, ferritin, Lactate Dehydrogenase, Direct Coombs Test, bone marrow biopsy, D-dimer, fibrinogen, partial thromboplastin time, ADAMTS13, HBV, HCM, HIV, parvovirus, anticardiolipin (ACA), antinuclear (ANA), anti-RNP, anti-Ro, anti-DNAds, anti-LKM, anti-MPO (p-ANCA), anti-PR3 (c-Anca), anti-smooth muscle antibodies; C3, C4, IgA, Ig G, IgM	Severe anemia (Hb 6.6–8 g/dL); severe thrombocytopenia (PCT 5–15 × 10^9^/L); slightly low levels of vitamin B12	Dexamethasone; RBc; vitamin B12; folic acid
34 WG	CBC	Moderate anemia (Hb 8.5 g/dL); severe thrombocytopenia (PCT 16 × 10^9^/L)	Dexamethasone (gradually tapered); IVIG (perioperative); leucocyte apheresis concentrate
Post- delivery	CBC	Severe/moderate anemia (Hb 6.7–9.7 g/dL); severe thrombocytopenia (PCT 18–26 × 10^9^/L)	RBc; vitamin B12; folic acid
**POSTPARTUM** **FOLLOW-UP** (3 months)	CBC	Pancytopenia (Hb 10.8 g/dL, PCT 20 × 10^9^/L, L = 3.32 × 10^9^/L)	

## Data Availability

No new data were created or analyzed in this study. Data sharing is not applicable to this article.
